# Efficacy and safety of isatuximab monotherapy to treat relapsed or refractory multiple myeloma: a pooled analysis of clinical trials

**DOI:** 10.1007/s00277-025-06343-9

**Published:** 2025-04-21

**Authors:** Meletios Dimopoulos, Kazutaka Sunami, Xavier Leleu, Ravi Vij, Cristina Gasparetto, Kenshi Suzuki, Sandrine Macé, Keisuke Tada, Mutsumi Hirakawa, Shinsuke Iida

**Affiliations:** 1 Department of Clinical Therapeutics, School of Medicine, National and Kapodistrian University of Athens, Athens, Greece; 2 Department of Medicine, Korea University, Seoul, South Korea; 3 Department of Hematology, NHO Okayama Medical Center, Okayama, Japan; 4 CIC 1082, U1313, CHU, University, Poitiers, France; 5 Division of Medical Oncology, Washington University in St. Louis, St. Louis, MO USA; 6 Hematologic Malignancies and Cellular Therapy, Duke University Medical Center, Durham, NC USA; 7 Myeloma/Amyloidosis Center, Japanese Red Cross Medical Center, Tokyo, Japan; 8 Research and Development, Sanofi, Paris, France; 9 Research and Development, Sanofi K.K., Tokyo, Japan; 10 Oncology Medical, Sanofi K.K., Tokyo, Japan; 11 Department of Hematology and Oncology, Nagoya City University Institute of Medical and Pharmaceutical Sciences, Kawasaki 1, Mizuno-cho, Mizuno-ku, Nagoya, Aichi 467-8601 Japan

**Keywords:** Dexamethasone, Isatuximab, Relapsed or refractory multiple myeloma, Pooled analysis

## Abstract

This pooled analysis of phase 1 and 2 clinical trials evaluated the efficacy and safety of isatuximab as monotherapy in individuals with relapsed or refractory multiple myeloma (RRMM) who had previously received a median of 4.0 lines of therapy; safety data for individuals on isatuximab plus dexamethasone has also been evaluated. The efficacy analysis (*n* = 167) showed that isatuximab 20 mg/kg monotherapy was effective in the treatment of individuals with RRMM, with an overall response rate (ORR) of 26.3%, a median progression-free survival (PFS) of 5.6 months and a median overall survival (OS) of 20.2 months. Isatuximab demonstrated rapid and sustained efficacy when used as monotherapy, with a median time to response of 1.0 month and a median duration of response of 10.3 months. The safety analysis, which included 477 participants from four clinical trials, showed that isatuximab had an acceptable safety profile that was consistent across all the dose groups examined. The most common treatment-emergent adverse events were infusion reactions (in 45.7% of participants), most of which were of grade 1 or 2 severity and occurred during the first infusion. Overall, no safety concerns were identified. In addition, participants aged ≥ 75 years had higher ORR and longer PFS and OS than those aged < 65 years, and since the proportions of patients with an Eastern Cooperative Oncology Group performance status of 2 did not vary significantly by age group, it appears that age does not affect the efficacy and safety of isatuximab treatment in RRMM. Further research is warranted to elaborate upon these findings.

**Supplementary Information:**

The online version contains supplementary material available at 10.1007/s00277-025-06343-9.

## Introduction

Multiple myeloma is the second most common haematological malignancy, with an age-standardised incidence of approximately 5 cases per 100,000 individuals [1, 2]. The median age at diagnosis is approximately 65 years, with < 3% of cases being < 40 years old [1–3]. Despite improvements in survival following the introduction of immunomodulatory drugs (IMiDs) and proteasome inhibitors (PIs), multiple myeloma remains incurable [4]. Therefore, the goal of treatment is to prolong survival and maintain quality of life [5].

Isatuximab is an immunoglobulin G1 (IgG1) monoclonal antibody against CD38, which is approved for the treatment of adults with relapsed or refractory multiple myeloma (RRMM) in combination with pomalidomide and dexamethasone, and carfilzomib and dexamethasone in Europe, Japan and the United States (US) [6], and in combination with bortezomib, lenalidomide, and dexamethasone in the US [7]. Uniquely, isatuximab is also approved as monotherapy for individuals with RRMM in Japan [8]. Employing isatuximab as monotherapy can be useful in patients with heavily pretreated RRMM [9], including those with a high cytogenetic risk [10]. Here, we present the efficacy and safety results of a pooled analysis of clinical trials of isatuximab monotherapy and in combination with dexamethasone in individuals with RRMM who received ≥ 3 previous lines of therapy or were refractory to a PI and an IMiD.

## Methods

### Pooled analysis design and clinical trial selection

For this analysis, data from all individuals who participated in Sanofi-sponsored clinical trials of isatuximab (alone, or in combination solely with dexamethasone) that enrolled individuals with RRMM (including the TED10893 trial that enrolled individuals with CD38-positive haematopoietic neoplasms) were pooled (Supplementary Fig. S1). A clinical trial was included if at least a part of the trial had been completed by the cut-off date and a clinical study report was available.

Based on the above criteria, data from the TED10893 (ClinicalTrials.gov identifier, NCT01084252) and TED14095 (NCT02812706; ISLANDs) were included in the efficacy analysis (Table 1). Demographic and baseline characteristics of individuals and efficacy outcomes were assessed. The efficacy analysis was restricted to data from individuals who had received isatuximab 20 mg/kg once weekly (QW) or once every 2 weeks (Q2W), and whose treatment responses were determined by Independent Review Committee assessment (i.e. TED10893 and TED14095). The 20 mg/kg isatuximab dose was determined from exposure–response analyses in the TED10893, TED14095 and TED14154 (Part B) trials, based on logistic regression using pharmacokinetic parameters as predictors of treatment response and response rates. Based on this logistic regression model, the probability of a trial reaching the target overall response rate (ORR) of ≥ 30% (set probability of success) was higher for 20 mg/kg QW/Q2W than for 10 mg/kg QW/Q2W, based on a clinical trial simulation.

**Table 1:** Details of selected clinical trials evaluating safety, pharmacokinetics, and efficacy of isatuximab

Trial (ClinicalTrials.gov identifier)	Study design	Study population	Treatment	Data cut-off date
TED10893 (NCT01084252) [13, 22]	Phase 1/2, dose-escalation and expansion	Phase 1: Individuals with CD38-positive hematopoietic neoplasms Phase 2: Individuals with RRMM	Phase 1: isatuximab ≤ 1 to 20 mg/kg QW or Q2W	–
			Phase 2, Stage 1: isatuximab 3 mg/kg Q2W, 10 or 20 mg/kg Q2W or Q2W/Q4W	9 December 2016
			Phase 2, Stage 2: isatuximab 20 mg/kg QW or QW/Q2W ± dexamethasone 40 mg/day^a^ on Days 1, 8, 15 and 22 of each 28-day cycle	21 January 2019
TED14095 (NCT02812706; ISLANDs) [10]	Phase 1/2, single-arm, open-label	Japanese individuals with RRMM	Phase 1: isatuximab 10 or 20 mg/kg QW/Q2W Phase 2: isatuximab 20 mg/kg QW/Q2W	10 December 2019
TED14154 (NCT02514668) [21]	Phase 1, open-label, dose-escalation	Individuals with RRMM	Part A: isatuximab 10 or 20 mg/kg QW/Q2W	6 July 2017
			Part B: isatuximab 20 mg/kg QW/Q2W	15 October 2018^b^
TCD14906 (NCT03194867) [23]	Phase 1/2, randomised, open-label	Individuals with RRMM who had received ≥ 3 prior lines of therapy	Arm A: isatuximab 10 mg/kg QW/Q2W + cemi 250 mg Q2W Arm B: isatuximab 10 mg/kg QW/Q2W + cemi Q4W Arm C: isatuximab 10 mg/kg QW/Q2W alone^c^	14 February 2020

^a^Patients aged ≥ 75 years were administered dexamethasone 20 mg/day

^b^Last participant completed the study

^c^Only participants in the active comparator arm were included in the present analysis

cemi, cemiplimab; QW, once weekly; Q2W, once every 2 weeks; Q4W, once every 4 weeks; QW/Q2W, once weekly followed by once every 2 weeks; RRMM, relapsed or refractory multiple myeloma

Data from the following trials were included in the safety analysis (Table 1): TED10893 (ClinicalTrials.gov identifier, NCT01084252), TED14095 (NCT02812706; ISLANDs), TED14154 (NCT02514668) and TCD14906 (NCT03194867). Demographic and baseline characteristics of individuals, prior and concomitant medications, medical history, extent of exposure and adverse events (AEs, including infusion reactions, laboratory findings, vital signs and anti-drug antibodies [ADAs]) were assessed. Two treatment groups were analysed: (1) isatuximab as monotherapy and (2) isatuximab + dexamethasone as part of the drug regimen. Data on the use of corticosteroids (dexamethasone or prednisone) were irrespective of whether this occurred as part of the trial drug regimen or as premedication.

All the trials included in this analysis were conducted in accordance with the Declaration of Helsinki, Good Clinical Practice and relevant guidelines. The protocols were approved by the appropriate individual ethics committees/institutional review boards for each trial, and all patients provided informed consent for participation.

### Endpoints

Efficacy endpoints were ORR, clinical benefit rate (CBR), clinical progression (CP) status, progression-free survival (PFS), overall survival (OS) and duration of response (DOR). ORR was defined as the proportion of participants who achieved complete response (CR), very good partial response (VGPR) or partial response (PR). CBR was defined as the proportion of participants who achieved CR, VGPR, PR or minimal response (MR). CP was based on three of the International Myeloma Working Group (IMWG) uniform response criteria for clinical relapse, whereby patients meeting any one of the following IMWG criteria during screening were classified as having CP: soft tissue plasmacytoma or occurrence of bone lesions; hypercalcemia (> 11.5 mg/dL; >2.875 mM/L); haemoglobin (Hb) < 10 g/dL [11]. Patients not meeting any of these criteria were classified as having non-CP status. Treatment responses (including renal response) were classified according to the IMWG criteria [12].

Safety endpoints were the incidence of treatment-emergent AEs (TEAEs), infusion reactions, respiratory TEAEs (lower respiratory tract TEAEs and respiratory infections), neutropenia and neutropenic complications, infections, thrombocytopenia and haemorrhage and ADAs. TEAEs were reported by system organ class (SOC) and preferred term (PT) using the Medical Dictionary for Regulatory Activities (MedDRA), version 22.1. AEs and laboratory abnormalities were graded according to the National Cancer Institute’s Common Terminology Criteria for Adverse Events (NCI-CTCAE), version 4.03.

### Statistical analysis

Descriptive statistics, including the number of observations, mean, standard deviation (SD), median, minimum and maximum for continuous variables, and the frequency and proportion for categorical variables, were calculated. Proportions were calculated using the number of participants with non-missing data as a denominator. PFS, OS and DOR were estimated using the Kaplan–Meier method and 95% confidence intervals (CIs) were calculated using log-log transformation of the survival function and the methods of Brookmeyer and Crowley. Multivariate analyses were conducted for the ORR (logistic regression), PFS and OS (Cox regression) to identify prognostic factors using a stepwise selection procedure with a 15% significance level for removing or adding effects; odds ratios (ORs) and 95% CIs were calculated for the ORR, and hazard ratios (HRs) and 95% CIs were calculated for PFS and OS. Statistical analyses were conducted using SAS software, version 9.4 or higher (SAS Institute, Cary, North Carolina, USA).

## Results

### Baseline characteristics – efficacy analysis set

A total of 167 participants who had received isatuximab 20 mg/kg as monotherapy were included in the efficacy analysis from TED14095 and TED10893 (Table 2). The median age of participants was 68.0 years, with most having an Eastern Cooperative Oncology Group performance status (ECOG PS) of 0 (43.7%) or 1 (47.3%), and 75.4% possessing normal renal function.

**Table 2:** Baseline characteristics of participants who received isatuximab 20 mg/kg as monotherapy (efficacy analysis population)

	TED14095 Phase 1 and Phase 2 (*n* = 33)	TED10893 Phase 2		All (*n* = 167)
		Stage 1 (*n* = 25)	Stage 2 (*n* = 109)	
Age, years, median (range)	72.0 (48–82)	59.0 (48–85)	68.0 (37–84)	68.0 (37–85)
Age group, n (%)				
< 65 years	6 (18.2)	16 (64.0)	44 (40.4)	66 (39.5)
65–74 years	15 (45.5)	7 (28.0)	44 (40.4)	66 (39.5)
≥ 75 years	12 (36.4)	2 (8.0)	21 (19.3)	35 (21.0)
Male sex, n (%)	19 (57.6)	12 (48.0)	51 (46.8)	82 (49.1)
Weight, kg, mean (SD)	55.3 (9.8)	73.9 (17.3)	74.5 (15.5)	70.6 (16.6)
ECOG PS, n (%)				
0	17 (51.5)	8 (32.0)	48 (44.0)	73 (43.7)
1	11 (33.3)	14 (56.0)	54 (49.5)	79 (47.3)
2	5 (15.2)	3 (12.0)	7 (6.4)	15 (9.0)
Renal response status, n (%)				
≥ 50 mL/min/1.73m^2^	27 (81.8)	21 (84.0)	78 (71.6)	126 (75.4)
≥ 30–50 mL/min/1.73m^2^	6 (18.2)	4 (16.0)	22 (20.2)	32 (19.2)
≥ 15–30 mL/min/1.73m^2^	–	–	3 (2.8)	3 (1.8)
< 15 mL/min/1.73m^2^	–	–	–	–
Missing	–	–	6 (5.5)	6 (3.6)

ECOG PS, Eastern Cooperative Oncology Group Performance Status; eGFR, estimated glomerular filtration rate; SD, standard deviation

### Efficacy

**Fig. 1:**
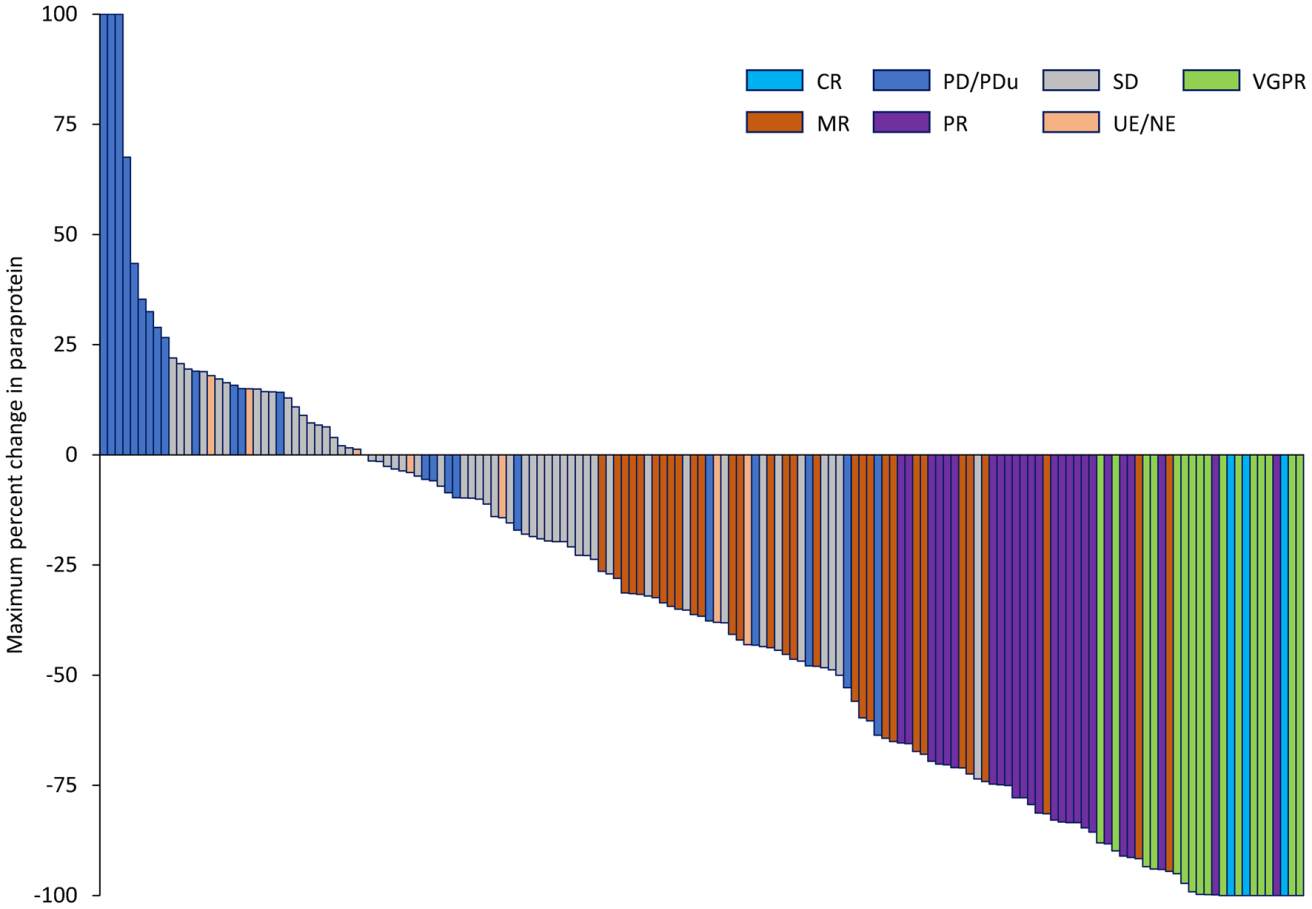
Waterfall plot of the maximum percent change in M-protein in the efficacy analysis population. CR, complete response; M-protein, non-functional intact immunoglobulins or immunoglobulin chains; MR, minimal response; NE, not evaluated; PD, progressive disease; PDu, unconfirmed progressive disease; PR, partial response; SD, stable disease; UE, unevaluable; VGPR, very good partial response

In the overall efficacy analysis population, the median duration of follow-up was 4.5 (range 0.0–29.8) months, the ORR was 26.3% (95% CI 19.8, 33.7; Table 3), and median time to response was 1.0 (range 0.9–9.1) month. The rate of VGPR or better response was 11.4% (95% CI 7.0, 17.2) and the CBR was 44.3% (95% CI 36.6, 52.2). Sixty-one participants (38.9%) had a ≥ 50% decrease in paraprotein and 24 (15.3%) had a ≥ 90% decrease in paraprotein (Fig. 1).

**Table 3:** Summary of response to isatuximab treatment by independent review committee decision in the efficacy analysis population (*n* = 167)

	TED14095 Phase 1 and Phase 2 (*n* = 33)	TED10893 Phase 2		All (*n* = 167)
		Stage 1 (*n* = 25)	Stage 2 (*n* = 109)	
Best response, n (%)				
CR	3 (9.1)	–	–	3 (1.8)
VGPR	4 (12.1)	2 (8.0)	10 (9.2)	16 (9.6)
PR	5 (15.2)	4 (16.0)	16 (14.7)	25 (15.0)
MR	6 (18.2)	3 (12.0)	21 (19.3)	30 (18.0)
Stable disease	7 (21.2)	12 (48.0)	35 (32.1)	54 (32.3)
PD	4 (12.1)	3 (12.0)	9 (8.3)	16 (9.6)
PDu	2 (6.1)	–	8 (7.3)	10 (6.0)
Not evaluable	2 (6.1)	1 (4.0)	10 (9.2)	13 (7.8)
ORR, n (%)	12 (36.4)	6 (24.0)	26 (23.9)	44 (26.3)
(95% CI)	(20.4, 54.9)	(9.4, 45.1)	(16.2, 33.0)	(19.8, 33.7)
VGPR or better, n (%)	7 (21.2)	2 (8.0)	10 (9.2)	19 (11.4)
(95% CI)	(9.0, 38.9)	(1.0, 26.0)	(4.5, 16.2)	(7.0, 17.2)
CBR, n (%)	18 (54.5)	9 (36.0)	47 (43.1)	74 (44.3)
(95% CI)	(36.4, 71.9)	(18.0, 57.5)	(33.7, 53.0)	(36.6, 52.2)

CI, confidence interval; CBR, clinical benefit rate; CR, complete response; MR, minimal response; ORR, overall response rate; PD, progressive disease; PDu, unconfirmed progressive disease; PR, partial response; VGPR, very good partial response

**Fig. 2:**
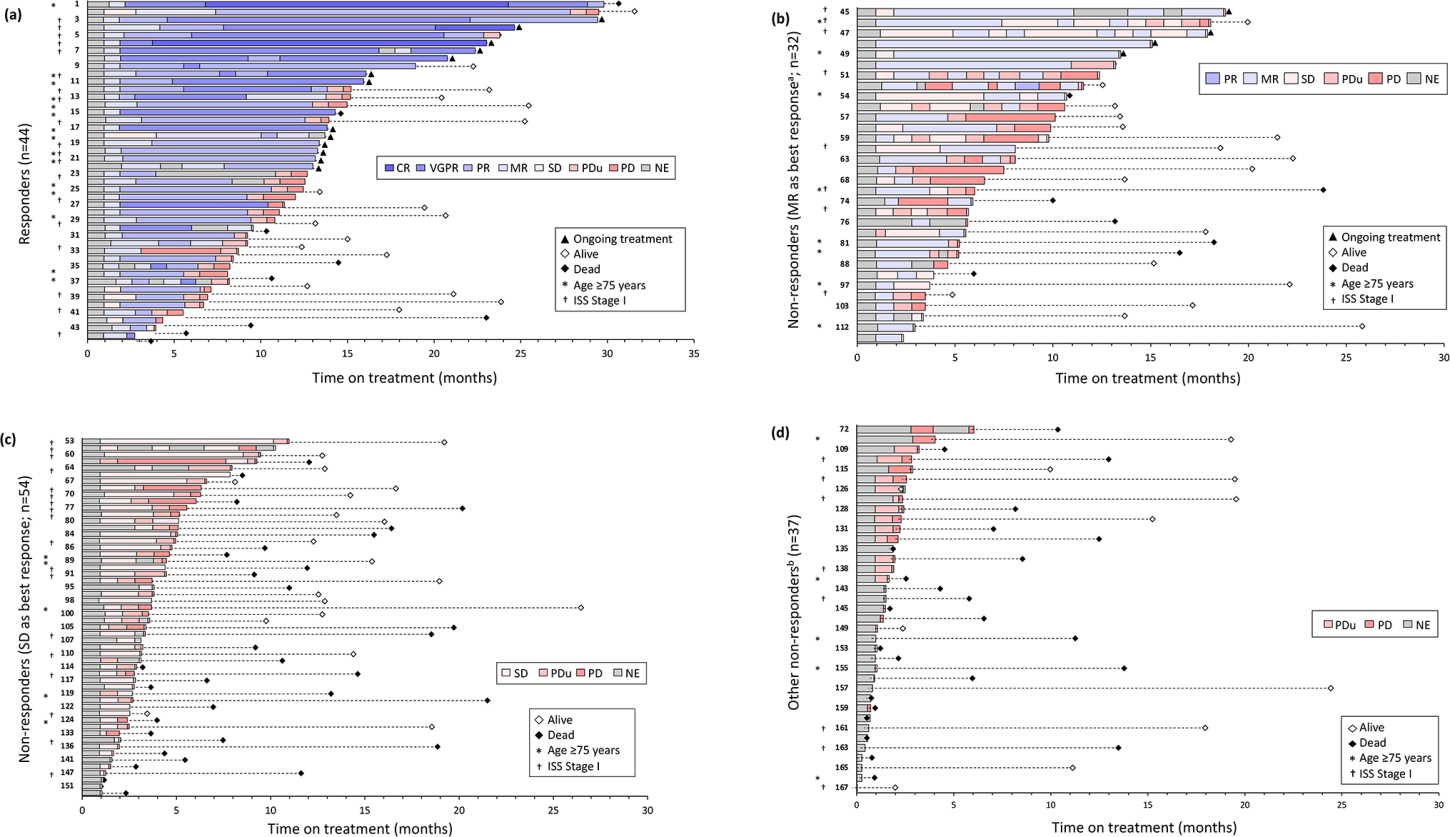
Swimmer plots of (**a**) participants with response (*n* = 44), (**b**) non-responders with minimal response as best response^a^ (*n* = 32), (**c**) non-responders with stable disease as best response (*n* = 54), and (**d**) other non-responders^b^ to isatuximab therapy in the efficacy analysis population. ^a^One participant was evaluated as PR once, but was classified as a “non-responder” based on IMWG criteria (i.e. two consecutive PR evaluations required to be classified as a “responder”). ^b^Included non-responders with PD, PDu, or NE as best response. CR, complete response; ISS, International Staging System; MR, minimal response; NE, not evaluated; PD, progressive disease; PDu, unconfirmed progressive disease; PR, partial response; SD, stable disease; UE, unevaluable; VGPR, very good partial response

**Fig. 3:**
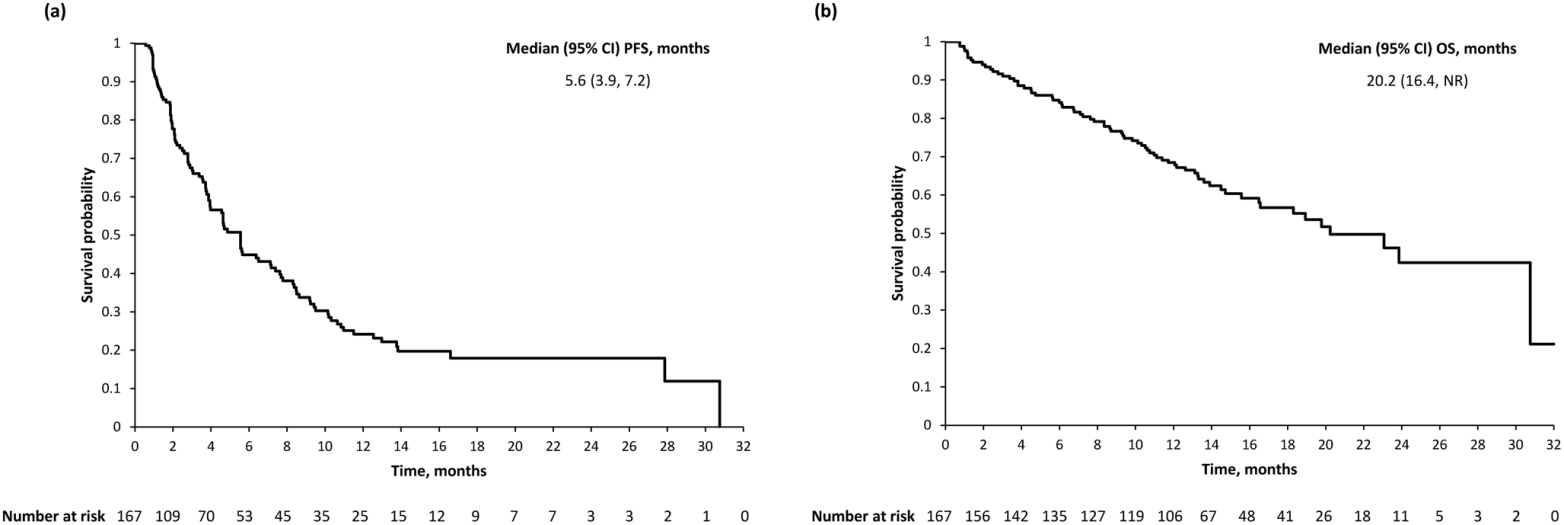
Kaplan–Meier curves of (**a**) progression-free survival and (**b**) overall survival in participants included in the efficacy analysis population CI, confidence interval; NR, not reached; OS, overall survival; PFS, progression-free survival

Of the 167 patients on isatuximab alone, there were 44 responders (Supplementary Table S1; Fig. 2A); of the non-responders, 32 had minimal response as the best response and 54 had stable disease as the best response (Fig. 2B-D). The median DOR was 10.3 (95% CI 8.28, 27.86) months and median PFS was 5.6 (95% CI 3.9, 7.2) months (Fig. 3A). In the multivariate analysis for PFS (Supplementary Table S2), International Staging System (ISS) stage III, high cytogenetic risk and plasmacytomas at baseline were significantly negatively associated with PFS. The median OS was 20.2 (95% CI 16.5, not reached) months (Fig. 3B). A subgroup analysis revealed no difference in PFS in individuals with or without CP at baseline (unadjusted HR 0.72; 95% CI 0.48, 1.08; Supplementary Fig. S2).

In the multivariate analysis of OS, age, ECOG PS, ISS stage and baseline estimated glomerular filtration rate (eGFR) were significantly associated with OS (Supplementary Table S3). In additional analyses conducted to confirm ECOG PS 2 status by age group, the proportions of patients were consistent among age groups, at 7.56% (5/66), 10.6% (7/66) and 8.5% (3/35) in the < 65-year, 65–74-year and ≥ 75-year age groups, respectively (*p* = 0.73). A subgroup analysis revealed no difference in OS in individuals with or without CP at baseline (unadjusted HR 0.84; 95% CI 0.513, 1.377; Supplementary Fig. S3).

Among the nine participants with baseline eGFR < 40 mL/min/1.73 m^2^, the ORR was 22.2%. The median PFS and OS were 2.1 (95% CI 1.9, not reached) months and 13.9 (95% CI 4.1, not reached) months, respectively (Supplementary Table S4). Renal response (CR or MR) was also reported in patients with different baseline eGFRs (< 50 mL/min/1.73 m^2^ or ≥ 15–30 mL/min/1.73 m^2^; Supplementary Table S5).

### Baseline characteristics – safety analysis set

A total of 477 participants who received isatuximab were included in the safety analysis (Table 4); data from two individuals with chronic lymphocytic leukaemia and three with non-Hodgkin’s lymphoma who participated in Phase 1 of the TED10893 trial were included. Of these, 47.6% (227/477) received isatuximab 20 mg/kg without dexamethasone and 11.5% (55/477) received isatuximab 20 mg/kg with dexamethasone; the remaining participants received ≤ 10 mg/kg isatuximab without dexamethasone. The median age of participants included in the safety analysis was 66.0 years.

**Table 4:** Baseline characteristics of participants included in the safety analysis population

	Isatuximab < 10 mg/kg (*n* = 48)	Isatuximab 10 mg/kg (*n* = 147)	Isatuximab 20 mg/kg (*n* = 227)	Isatuximab 20 mg/kg + dexamethasone (*n* = 55)	All (*n* = 477)
Age, years, median (range)	63.5 (41–85)	65.0 (38–83)	68.0 (37–85)	66.0 (42–85)	66.0 (37–85)
Age group, n (%)					
< 65	26 (54.2)	73 (49.7)	88 (38.8)	19 (34.5)	206 (43.2)
65–74 years	15 (31.3)	61 (41.5)	90 (39.6)	24 (43.6)	190 (39.8)
≥ 75 years	7 (14.6)	13 (8.8)	49 (21.6)	12 (21.8)	81 (17.0)
Weight, kg, mean (SD)	81.40 (22.82)	79.32 (21.08)	71.69 (17.23)	73.72 (19.92)	75.25 (19.72)
Male sex, n (%)	28 (58.3)	85 (57.8)	113 (49.8)	29 (52.7)	255 (53.5)
ECOG PS, n (%)					
0	9 (18.8)	28 (19.0)	86 (37.9)	27 (49.1)	150 (31.4)
1	29 (60.4)	106 (72.1)	113 (49.8)	22 (40.0)	270 (56.6)
2	10 (20.8)	13 (8.8)	27 (11.9)	6 (10.9)	56 (11.7)
3	–	–	1 (0.4)	–	1 (0.2)
ISS stage, n (%)					
I	14 (29.2)	40 (27.2)	77 (33.9)	15 (27.3)	146 (30.6)
II	15 (31.3)	58 (39.5)	74 (32.6)	20 (36.4)	167 (35.0)
III	13 (27.1)	46 (31.3)	75 (33.0)	20 (36.4)	154 (32.3)
Unknown/missing	6 (12.5)	3 (2.0)	1 (0.4)	–	10 (2.1)
Bone marrow plasma cells, %, mean (SD)	43.73 (31.37)	34.84 (30.74)	30.56 (27.05)	30.69 (23.76)	33.38 (28.67)
Cytogenetics by FISH, n (%)					
Standard risk	21 (43.8)	32 (23.7)	106 (49.8)	24 (43.6)	183 (40.6)
High risk	9 (18.8)	34 (25.2)	52 (24.4)	12 (21.8)	107 (23.7)
Unknown/missing	18 (37.5)	69 (51.1)	55 (25.8)	19 (34.5)	161 (35.7)
Hepatic function,^a^ n (%)					
Normal	38 (79.2)	127 (86.4)	179 (78.9)	42 (76.4)	386 (80.9)
Mild impairment	10 (20.8)	20 (13.6)	21 (9.3)	7 (12.7)	58 (12.2)
Moderate impairment	–	–	1 (0.4)	–	1 (0.2)
Severe impairment	–	–	–	–	–
Renal function,^b^ n (%)					
Normal	13 (27.1)	19 (12.9)	45 (19.8)	13 (23.6)	90 (18.9)
Mild impairment	17 (35.4)	59 (40.1)	86 (37.9)	23 (41.8)	185 (38.8)
Moderate impairment	13 (27.1)	51 (34.7)	63 (27.8)	13 (23.6)	140 (29.4)
Severe impairment	1 (2.1)	6 (4.1)	5 (2.2)	4 (7.3)	16 (3.4)
End-stage renal disease	1 (2.1)	1 (0.7)	–	–	2 (0.4)
Time since last dose of antimyeloma therapy, months, mean (SD)	2.4 (2.3)	3.6 (6.5)	2.9 (5.3)	4.7 (9.5)	3.3 (6.1)
Number of prior lines of therapy, median (range)	5.0 (1–13)	5.0 (1–14)	4.0 (2–14)	4.0 (2–10)	4.0 (1–14)
Main previous treatments, n (%)					
Alkylating agent	47 (97.9)	140 (95.2)	219 (96.5)	53 (96.4)	459 (96.2)
PI agent	44 (91.7)	146 (99.3)	227 (100)	55 (100)	472 (99.0)
IMiD agent	45 (93.8)	146 (99.3)	225 (99.1)	55 (100)	471 (98.7)
Lenalidomide	43 (89.6)	139 (94.6)	199 (87.7)	43 (78.2)	424 (88.9)
Pomalidomide	22 (45.8)	86 (58.5)	126 (55.5)	25 (45.5)	259 (54.3)
Monoclonal antibody	1 (2.1)	12 (8.2)	45 (19.8)	3 (5.5)	61 (12.8)
Daratumumab	–	10 (6.8)	32 (14.1)	–	42 (8.8)
Elotuzumab	1 (2.1)	2 (1.4)	13 (5.7)	3 (5.5)	19 (4.0)
Corticosteroid	46 (95.8)	147 (100)	226 (99.6)	55 (100)	474 (99.4)

^a^Hepatic function was defined as follows: normal: total bilirubin ≤ ULN and AST ≤ ULN; mild: total bilirubin > ULN to ≤ 1.5 × ULN and any AST or total bilirubin ≤ ULN and AST > ULN; moderate: total bilirubin > 1.5 × ULN to ≤ 3 × ULN and any AST; severe: total bilirubin > 3 × ULN and any AST

At baseline, 31.4% of participants had an ECOG PS of 0 and 56.6% had an ECOG PS of 1. Roughly equal proportions of participants had Stage I (30.6%), Stage II (35.0%) and Stage III (32.3%) disease according to the ISS. The mean percentage of bone marrow plasma cells was 33.4%. Fluorescence in situ hybridization (FISH) analysis classified 23.7% with high-risk disease, defined as presence of either of t(4;14), t(14;16), or del(17p). Sixteen participants (3.4%) had severe renal impairment.

### Exposure

The median number of treatment cycles started was 4.0 across all the dose groups and was higher in participants who received isatuximab 20 mg/kg with dexamethasone than in the other groups (7.0 vs. 2.0–4.0). The median duration of isatuximab treatment was 15.7 weeks across all dose groups and was longer in participants who received isatuximab 20 mg/kg with dexamethasone than in other dose groups (30.0 vs. 5.9–16.0 weeks) (Table 5). The most common reason for discontinuation of study treatment was disease progression (73.8%), followed by AEs (6.7%).

**Table 5:** Study treatment exposure in the safety analysis population

	Isatuximab < 10 mg/kg (*n* = 48)	Isatuximab 10 mg/kg (*n* = 147)	Isatuximab 20 mg/kg (*n* = 227)	Isatuximab 20 mg/kg + dexamethasone (*n* = 55)	All (*n* = 477)
Number of cycles started by participant					
Mean (SD)	3.9 (5.4)	5.8 (5.8)	6.6 (6.1)	9.0 (6.3)	6.3 (6.1)
Median (range)	2.0 (1–28)	4.0 (1–28)	4.0 (1–33)	7.0 (1–22)	4.0 (1–33)
Duration of isatuximab exposure, weeks					
Mean (SD)	15.4 (22.3)	23.1 (24.1)	26.1 (25.3)	36.3 (26.4)	25.3 (25.2)
Median (range)	5.9 (2.0–116.3)	14.4 (2.0–120.4)	16.0 (1.0–131.4)	30.0 (1.0–91.9)	15.7 (1.0–131.4)
Ongoing treatment, n (%)	1 (2.1)	14 (9.5)	20 (8.8)	15 (27.3)	50 (10.5)
Duration of isatuximab exposure, n (%)					
At least 6 months	7 (14.6)	42 (28.6)	78 (34.4)	31 (56.4)	158 (33.1)
At least 12 months	4 (8.3)	15 (10.2)	31 (13.7)	21 (38.2)	71 (14.9)
At least 18 months	1 (2.1)	5 (3.4)	9 (4.0)	3 (5.5)	18 (3.8)
More than 24 months	1 (2.1)	3 (2.0)	4 (1.8)	–	8 (1.7)
Relative dose intensity, %					
Mean (SD)	129.1 (87.5)	94.67 (17.0)	95.4 (16.7)	94.7 (18.2)	98.5 (33.5)
Median (range)	100.0 (80.2–525.6)	99.6 (14.0–141.3)	99.2 (1.5–123.1)	97.6 (9.4–112.5)	99.5 (1.5–525.6)
Main reasons for definitive study treatment discontinuation, n (%)					
AE	1 (2.1)	6 (4.1)	20 (8.8)	5 (9.1)	32 (6.7)
PD	42 (87.5)	116 (78.9)	161 (70.9)	33 (60.0)	352 (73.8)
Poor compliance with protocol	–	–	1 (0.4)	–	1 (0.2)
Withdrawal by participant	–	1 (0.7)	–	–	1 (0.2)
Other reason	4 (8.3)	10 (6.8)	25 (11.0)	2 (3.6)	41 (8.6)

AE, adverse event; PD, progressive disease; SD, standard deviation

Isatuximab therapy was accompanied by prophylaxis that was administered in anticipation of an infusion reaction. Before the first infusion, > 95.0% of participants received prophylaxis, including paracetamol, corticosteroids and histamine 1 antagonists. Corticosteroids (dexamethasone, prednisone) were administered as premedication to 99.4% of patients. Infusion reaction prophylaxis was administered in > 92.0% of participants before the second and subsequent infusions of isatuximab. Overall, isatuximab was administered in combination with prophylactic or therapeutic systemic antivirals or systemic antibacterials in 67.3% and 69.8% of participants, respectively.

### Safety

In the safety analysis set, TEAEs of any grade occurred in 453 participants (95.0%) and TEAEs of grade ≥ 3 occurred in 260 participants (54.5%; Table 6). The incidence of TEAEs of any grade and TEAEs of grade ≥ 3 was similar across dose groups.

**Table 6:** Summary of treatment-emergent adverse events (TEAEs) that occurred in patients included in the safety analysis population

TEAEs, *n* (%)	Isatuximab < 10 mg/kg (*n* = 48)	Isatuximab 10 mg/kg (*n* = 147)	Isatuximab 20 mg/kg (*n* = 227)	Isatuximab 20 mg/kg + dexamethasone (*n* = 55)	All (*n* = 477)
Any TEAEs					
Any grade	47 (97.9)	147 (100)	208 (91.6)	51 (92.7)	453 (95.0)
Grade ≥ 3	30 (62.5)	82 (55.8)	115 (50.7)	33 (60.0)	260 (54.5)
Any treatment-related TEAEs					
Any grade	28 (58.3)	108 (73.5)	141 (62.1)	40 (72.7)	317 (66.5)
Grade ≥ 3	4 (8.3)	20 (13.6)	28 (12.3)	10 (18.2)	62 (13.0)
Serious TEAEs, n (%)	20 (41.7)	64 (43.5)	94 (41.4)	25 (45.5)	203 (42.6)
Serious treatment-related TEAEs	2 (4.2)	9 (6.1)	21 (9.3)	5 (9.1)	37 (7.8)
TEAEs with a fatal outcome	4 (8.3)	5 (3.4)	18 (7.9)	4 (7.3)	31 (6.5)
Treatment-related TEAEs with a fatal outcome	–	–	2 (0.9)	–	2 (0.4)
TEAEs leading to definitive discontinuation	1 (2.1)	6 (4.1)	20 (8.8)	3 (5.5)	30 (6.3)
Isatuximab	–	–	–	1 (1.8)	1 (0.2)
Dexamethasone	–	–	–	2 (3.6)	2 (0.4)
≥ 1 infusion reaction	17 (35.4)	83 (56.5)	93 (41.0)	21 (38.2)	214 (44.9)
Worst grade of infusion reaction					
Grade 1	2 (4.2)	14 (9.5)	14 (6.2)	1 (1.8)	31 (6.5)
Grade 2	15 (31.3)	66 (44.9)	73 (32.2)	18 (32.7)	172 (36.1)
Grade ≥ 3	–	3 (2.0)	6 (2.6)	2 (3.6)	11 (2.3)
Timing of infusion reactions					
First infusion	15 (31.3)	81 (55.1)	90 (39.6)	21 (38.2)	207 (43.4)
Subsequent infusions	5 (10.4)	9 (6.1)	6 (2.6)	–	20 (4.2)

The most common TEAEs of any grade by SOC were infectious and parasitic diseases (57.2%; Table 7); by PT, they were infusion-related reactions (45.7%), fatigue (23.5%), diarrhoea (20.3%), nausea (18.0%) and upper respiratory infection (18.0%). The most common TEAEs of grade ≥ 3 by SOC were infectious and parasitic diseases (20.5%), while those by PT were anaemia (11.9%), pneumonia (7.5%), thrombocytopenia (5.7%), disease progression (4.2%) and neutropenia (3.4%). Of note, two participants (0.4%) reported hypogammaglobulinaemia (both grade < 3, in the 10 mg/kg group) and one participant (0.2%) developed cytomegalovirus infection (grade < 3, in the 20 mg/kg group).

**Table 7:** Treatment-emergent adverse events (TEAEs) of any grade with an incidence of ≥ 5% and of grade ≥ 3 with an incidence of ≥ 2% that occurred in patients included in the safety analysis population

TEAEs, *n* (%) Primary system-organ-class Preferred term	Isatuximab < 10 mg/kg (*n* = 48)		Isatuximab 10 mg/kg (*n* = 147)		Isatuximab 20 mg/kg (*n* = 227)		Isatuximab 20 mg/kg + dexamethasone (*n* = 55)		All (*n* = 477)	
	Any grade	Grade ≥ 3	Any grade	Grade ≥ 3	Any grade	Grade ≥ 3	Any grade	Grade ≥ 3	Any grade	Grade ≥ 3
Any class	47 (97.9)	30 (62.5)	147 (100.0)	82 (55.8)	208 (91.6)	115 (50.7)	51 (92.7)	33 (60.0)	453 (95.0)	260 (54.5)
Infectious and parasitic diseases	25 (52.1)	11 (22.9)	90 (61.2)	32 (21.8)	126 (55.5)	43 (18.9)	32 (58.2)	12 (21.8)	273 (57.2)	98 (20.5)
Upper respiratory tract infection	9 (18.8)	2 (4.2)	35 (23.8)	1 (0.7)	34 (15.0)	2 (0.9)	8 (14.5)	1 (1.8)	86 (18.0)	6 (1.3)
Pneumonia	4 (8.3)	2 (4.2)	17 (11.6)	17 (11.6)	18 (7.9)	13 (5.7)	7 (12.7)	4 (7.3)	46 (9.6)	36 (7.5)
Bronchitis	2 (4.2)	1 (2.1)	8 (5.4)	–	18 (7.9)	1 (0.4)	4 (7.3)	3 (5.5)	32 (6.7)	5 (1.0)
Epipharyngitis	1 (2.1)	–	7 (4.8)	–	13 (5.7)	–	7 (12.7)	–	28 (5.9)	–
Urinary tract infection	4 (8.3)	1 (2.1)	7 (4.8)	1 (0.7)	12 (5.3)	1 (0.4)	1 (1.8)	–	24 (5.0)	3 (0.6)
Sepsis	4 (8.3)	4 (8.3)	5 (3.4)	5 (3.4)	4 (1.8)	4 (1.8)	2 (3.6)	2 (3.6)	15 (3.1)	15 (3.1)
Blood and lymphatic system disorders	19 (39.6)	15 (31.3)	38 (25.9)	28 (19.0)	44 (19.4)	33 (14.5)	2 (3.6)	2 (3.6)	103 (21.6)	78 (16.4)
Anaemia	17 (35.4)	12 (25.0)	30 (20.4)	21 (14.3)	32 (14.1)	24 (10.6)	–	–	79 (16.6)	57 (11.9)
Thrombocytopenia	6 (12.5)	6 (12.5)	8 (5.4)	7 (4.8)	15 (6.6)	13 (5.7)	1 (1.8)	1 (1.8)	30 (6.3)	27 (5.7)
Neutropenia	1 (2.1)	1 (2.1)	8 (5.4)	7 (4.8)	12 (5.3)	8 (3.5)	–	–	21 (4.4)	16 (3.4)
Metabolic and nutritional disorders	16 (33.3)	6 (12.5)	42 (28.6)	11 (7.5)	46 (20.3)	10 (4.4)	7 (12.7)	–	111 (23.3)	27 (5.7)
Loss of appetite	5 (10.4)	–	19 (12.9)	–	24 (10.6)	1 (0.4)	4 (7.3)	–	52 (10.9)	1 (0.2)
Mental disorders	8 (16.7)	1 (2.1)	25 (17.0)	–	31 (13.7)	2 (0.9)	17 (30.9)	3 (5.5)	81 (17.0)	6 (1.3)
Insomnia	3 (6.3)	–	15 (10.2)	–	11 (4.8)	–	14 (25.5)	1 (1.8)	43 (9.0)	1 (0.2)
Nervous system disorders	15 (31.3)	2 (4.2)	48 (32.7)	4 (2.7)	72 (31.7)	13 (5.7)	19 (34.5)	5 (9.1)	154 (32.3)	24 (5.0)
Headache	6 (12.5)	1 (2.1)	21 (14.3)	–	31 (13.7)	–	7 (12.7)	–	65 (13.6)	1 (0.2)
Dizziness	2 (4.2)	–	7 (4.8)	–	11 (4.8)	–	4 (7.3)	–	24 (5.0)	–
Respiratory, thoracic and mediastinal disorders	20 (41.7)	3 (6.3)	67 (45.6)	4 (2.7)	70 (30.8)	12 (5.3)	13 (23.6)	1 (1.8)	170 (35.6)	20 (4.2)
Cough	8 (16.7)	–	32 (21.8)	–	19 (8.4)	–	6 (10.9)	–	65 (13.6)	–
Dyspnoea	5 (10.4)	–	17 (11.6)	2 (1.4)	12 (5.3)	3 (1.3)	3 (5.5)	–	37 (7.8)	5 (1.0)
Gastrointestinal disorders	25 (52.1)	3 (6.3)	78 (53.1)	6 (4.1)	87 (38.3)	11 (4.8)	23 (41.8)	4 (7.3)	213 (44.7)	24 (5.0)
Diarrhoea	9 (18.8)	–	35 (23.8)	2 (1.4)	42 (18.5)	2 (0.9)	11 (20.0)	2 (3.6)	97 (20.3)	6 (1.3)
Nausea	9 (18.8)	–	43 (29.3)	–	27 (11.9)	1 (0.4)	7 (12.7)	–	86 (18.0)	1 (0.2)
Vomiting	3 (6.3)	–	25 (17.0)	1 (0.7)	23 (10.1)	1 (0.4)	–	–	51 (10.7)	2 (0.4)
Constipation	9 (18.8)	–	11 (7.5)	–	15 (6.6)	1 (0.4)	3 (5.5)	–	38 (8.0)	1 (0.2)
Musculoskeletal and connective tissue disorders	22 (45.8)	3 (6.3)	79 (53.7)	12 (8.2)	101 (44.5)	16 (7.0)	27 (49.1)	4 (7.3)	229 (48.0)	35 (7.3)
Back pain	4 (8.3)	1 (2.1)	29 (19.7)	5 (3.4)	40 (17.6)	5 (2.2)	9 (16.4)	–	82 (17.2)	11 (2.3)
Limb pain	4 (8.3)	–	13 (8.8)	–	15 (6.6)	1 (0.4)	9 (16.4)	2 (3.6)	41 (8.6)	3 (0.6)
Arthralgia	3 (6.3)	–	14 (9.5)	1 (0.7)	18 (7.9)	–	4 (7.3)	–	39 (8.2)	1 (0.2)
Bone pain	5 (10.4)	1 (2.1)	17 (11.6)	3 (2.0)	15 (6.6)	4 (1.8)	1 (1.8)	–	38 (8.0)	8 (1.7)
Musculoskeletal chest pain	5 (10.4)	–	7 (4.8)	1 (0.7)	13 (5.7)	2 (0.9)	4 (7.3)	–	29 (6.1)	3 (0.6)
Myalgia	1 (2.1)	–	10 (6.8)	–	12 (5.3)	0	3 (5.5)	1 (1.8)	26 (5.5)	1 (0.2)
Musculoskeletal pain	1 (2.1)	–	8 (5.4)	–	13 (5.7)	2 (0.9)	3 (5.5)	–	25 (5.2)	2 (0.4)
Renal and urinary tract disorders	6 (12.5)	3 (6.3)	22 (15.0)	7 (4.8)	22 (9.7)	9 (4.0)	3 (5.5)	2 (3.6)	53 (11.1)	21 (4.4)
Acute kidney injury	4 (8.3)	2 (4.2)	9 (6.1)	6 (4.1)	7 (3.1)	5 (2.2)	2 (3.6)	2 (3.6)	22 (4.6)	15 (3.1)
General and systemic disorders and administration site conditions	31 (64.6)	7 (14.6)	86 (58.5)	10 (6.8)	99 (43.6)	21 (9.3)	24 (43.6)	4 (7.3)	240 (50.3)	42 (8.8)
Fatigue	16 (33.3)	–	41 (27.9)	1 (0.7)	45 (19.8)	5 (2.2)	10 (18.2)	–	112 (23.5)	6 (1.3)
Fever	6 (12.5)	1 (2.1)	22 (15.0)	1 (0.7)	16 (7.0)	1 (0.4)	7 (12.7)	1 (1.8)	51 (10.7)	4 (0.8)
Peripheral oedema	4 (8.3)	–	15 (10.2)	–	14 (6.2)	1 (0.4)	5 (9.1)	–	38 (8.0)	1 (0.2)
Asthenia	2 (4.2)	1 (2.1)	11 (7.5)	1 (0.7)	15 (6.6)	1 (0.4)	6 (10.9)	1 (1.8)	34 (7.1)	4 (0.8)
Disease progression	5 (10.4)	5 (10.4)	2 (1.4)	2 (1.4)	11 (4.8)	11 (4.8)	2 (3.6)	2 (3.6)	20 (4.2)	20 (4.2)
Injury, poisoning and procedural complications	22 (45.8)	–	95 (64.6)	3 (2.0)	106 (46.7)	9 (4.0)	26 (47.3)	3 (5.5)	249 (52.2)	15 (3.1)
Infusion-associated reaction	19 (39.6)	–	84 (57.1)	3 (2.0)	94 (41.4)	6 (2.6)	21 (38.2)	2 (3.6)	218 (45.7)	11 (2.3)

Potential TEAEs of any grade occurred in 317 participants (66.5%) with grade ≥ 3 in 62 participants (13.0%). A total of 31 deaths (6.5%) occurred during isatuximab treatment across all dose groups. Causes of death were disease progression in 17 participants (3.6%), TEAEs in 13 (2.7%) and other causes in one (0.2%). TEAEs that led to death and occurred in > 1 participant were sepsis (*n* = 4, 0.8%), respiratory tract infection (*n* = 2, 0.4%), acute kidney injury (*n* = 2, 0.4%) and haemorrhage (*n* = 3, 0.6%). There were two TEAEs with fatal outcomes in the 20 mg/kg group (0.9%; Table 7); the causes of death were respiratory infection and sepsis (*n* = 1 each). Infusion reactions occurred in 214 participants (44.9%) across all dose groups. The most common grade of infusion reactions was grade 2 (36.1%), followed by grade 1 (6.5%), grade 3 (1.5%) and grade 4 (0.8%). Infusion reactions were more common during the first infusion (43.4%) than during subsequent infusions (4.2%).

A comparative analysis between the isatuximab monotherapy and isatuximab + dexamethasone groups was not conducted. However, a lower proportion of all-grade AEs were observed in the isatuximab monotherapy group (6.9%) versus the isatuximab + dexamethasone group (25.5%); a similar higher incidence was observed with insomnia AEs. There was also an increased incidence of gastrointestinal disorders in TED10893; however, the increasing trend was observed in Grade 3 or higher AEs in the dexamethasone group in the integrated analysis (4.7% vs. 7.3%). Although the incidence of hyperglycemia and cataracts was low overall, the higher incidences occurred with the addition of dexamethasone (1.2% vs. 5.5% and 1.7% vs. 5.5%, respectively). The incidence of Grade 3 or higher hypertension AEs were slightly higher in in the isatuximab + dexamethasone group (1.7% vs. 3.6%).

## Discussion

This pooled analysis of phase 1/2 clinical trials was conducted to evaluate the efficacy and safety of isatuximab treatment in individuals with RRMM, specifically focusing on patients who were on isatuximab monotherapy.

In this pooled analysis, monotherapy with isatuximab 20 mg/kg was effective in the treatment of participants with RRMM, demonstrating an ORR of 26.3%, a median PFS of 5.6 months and a median OS of 20.2 months. Multivariate subgroup analyses showed that participants aged ≥ 75 years had higher ORR and longer PFS and OS than those aged < 65 years. In addition, participants with a better prognosis (i.e. ISS Stage I, ECOG PS of 0 or 1 and standard-risk cytogenetics) had higher ORR and longer PFS and OS than those with a poorer prognosis (i.e. ISS Stage III, ECOG PS of 2 and high-risk cytogenetics). Interestingly, additional analyses that explored the proportions of patients with ECOG PS 2 by age group found no significant variation between the different cohorts, indicating that age does not affect the efficacy and safety of isatuximab treatment in RRMM. Analyses also determined that participants with an eGFR of ≥ 60 mL/min/1.73 m^2^ had longer OS than those with an eGFR of < 60 mL/min/1.73 m^2^. The marked discrepancy between PFS and the much longer OS in this pooled analysis has also been reported in previous clinical trials involving patients with RRMM: in the TED10893 trial, the median PFS was 4.9 (95% CI 3.9, 7.7) months and median OS was 18.9 (95% CI 3.6, 23.1) months with isatuximab monotherapy [13], while in a prespecified second analysis of the TED14095 trial conducted 20 months after the first dosing of the last participant, the median PFS was 5.6 (95% CI 3.8, 13.8) months, and the median OS was 30.8 (95% CI 23.9, not reached) months [14]. It is speculated that survival in the TED14095 trial may have been improved by the availability of many post-treatment facilities and other investigational drugs as treatment options.

The efficacy of isatuximab monotherapy was rapid and sustained, with a median time to response of 1.0 month and a median DOR of 10.3 months. These results compare favourably with the efficacy of daratumumab monotherapy, as demonstrated in the SIRIUS trial [15]. In SIRIUS, daratumumab monotherapy was associated with an ORR of 29.2% and a median PFS of 3.7 months, while median OS was not reached [15]. Although isatuximab and daratumumab are both monoclonal antibodies against CD38, there are several differences in the mechanisms of action of these drugs [16]. The binding site for isatuximab partially encompasses the catalytic site of CD38, while the binding site of daratumumab is located away from the catalytic site of CD38 [16]. As a result, isatuximab causes near-complete inhibition of CD38 activity, while daratumumab causes only partial inhibition [16].

Lenalidomide is another drug that may be used as monotherapy in patients with MM following an autologous stem cell transplant [17]. However, it appears to be associated with an increased risk of second primary malignancies [17]. In our analysis of pooled data from isatuximab trials, the incidence of second primary malignancies was 2.9% (14/477) [data not shown], which falls within the range of the natural incidence rates of such malignancies in patients with RRMM (1.7–6.6%) [18]. This suggests that the risk of second primary malignancies in patients with RRMM may be influenced by various factors, including advanced age, sun exposure, precancerous lesions, previous diseases, immunosuppression due to underlying disease, a history of radiation therapy and exposure to alkylating agents such as melphalan, as well as lenalidomide [18].

The safety analysis, which included 477 participants from four clinical trials, showed that isatuximab with or without dexamethasone had an acceptable safety profile, and that as monotherapy, had a consistent safety profile across all the dose groups examined. The most common TEAEs by PT were infusion reactions (in 45.7% of participants), most of which were of grade 1 or 2 severity and had occurred during the first infusion. The rate of infusion reactions with isatuximab in the present analysis was similar to that reported with daratumumab monotherapy in an open-label, randomised phase 2 SIRIUS trial in participants with RRMM (42%) [15] and in a *post hoc* pooled analysis of daratumumab monotherapy in participants with RRMM from both the SIRIUS trial and the open-label, non-randomised phase 2 GEN501 trial (48%) [19].

Infections were among the most common TEAEs in this pooled analysis, with upper respiratory tract infections and pneumonia occurring in 18.0% and 9.6% of the overall safety analysis population, respectively. As reported previously [1–3, 20], individuals with RRMM tended to be older, heavily pretreated and had suppressed immunity. Of note, participants in this pooled analysis had received a median of 4.0 prior lines of therapy, 4.4% of participants had neutropenia of any grade and 3.4% had neutropenia of grade ≥ 3.

Although isatuximab has demonstrated efficacy and safety in phase 2 trials in individuals with RRMM [10, 21–23], caution has been raised regarding the risk of infections and hypogammaglobulinaemia associated with novel immunotherapies [20]. In this pooled analysis, 98 participants (20.5%) had grade ≥ 3 infectious or parasitic diseases during isatuximab treatment, but only two participants (0.4%) had hypogammaglobulinaemia (both grade < 3, in the 10 mg/kg group) and one participant (0.4%) had grade < 3 cytomegalovirus infection in the 20 mg/kg group. Due to the tendency of individuals with RRMM to be older and/or have a lower performance status, it is crucial to consider the risk of infection associated with each medication in such individuals, and to take appropriate preventive measures. In this analysis, the impact of corticosteroids as premedication on infection risk could not be excluded. However, since patients using this regimen are likely to have a lower performance status, data on older patients and of corticosteroid-free treatment are required in future analyses.

In the MM-003 trial, which compared the efficacy and safety of pomalidomide (4 mg/day on days 1–21) plus low-dose dexamethasone (40 mg/day on days 1, 8, 15 and 22) with high-dose dexamethasone alone (40 mg/day on days 1–4, 9–12 and 17–20) in patients with RRMM, the median PFS was 4.0 (95% CI 3.6, 4.7) months for the pomalidomide plus low-dose dexamethasone arm versus 1.9 (95% CI 1.9, 2.2) months with high-dose dexamethasone [24]. In additional analyses adjusting for crossover of patients in the high-dose dexamethasone arm to pomalidomide plus low-dose dexamethasone in the MM-003 trial, the adjusted difference in median OS between patients in the pomalidomide plus low-dose dexamethasone and high-dose dexamethasone arms was 7.0 months (12.7 vs. 5.7 months, respectively) [25]. Synergistic activity of isatuximab in combination with dexamethasone was shown to improve response rate and PFS in clinical trials to treat newly-diagnosed MM [16] Because data are scant regarding the long-term administration of isatuximab with or without dexamethasone further studies are needed. Therefore, the addition of isatuximab to pomalidomide/dexamethasone regimens may improve outcomes in the RRMM patient population.

This analysis was based on pooled data from phase 1 and 2 clinical trials, and as such, has limitations. Firstly, the number of participants in some trials was small and the trials lacked comparator groups. In addition, as is the case in most clinical trials, participants were selected according to strict criteria, which may limit the generalisability of these findings. Furthermore, separate analyses of safety could have been conducted in the isatuximab monotherapy group versus the isatuximab + dexamethasone group.

In conclusion, this pooled analysis of industry-sponsored clinical trials provides valuable insights into the efficacy and safety of isatuximab monotherapy in the treatment of individuals with RRMM. The results show that isatuximab as monotherapy was efficacious in the treatment of individuals with RRMM, and that isatuximab had an acceptable safety profile when used alone and in combination with dexamethasone. Therefore, adding isatuximab to existing RRMM treatment regimens may improve outcomes, particularly in those who have been heavily pretreated for RRMM, are refractory to a PI and an IMiD, and even in individuals aged ≥ 75 years presenting with an ECOG PS of 2. Further research to confirm and expand on these findings is warranted.

## Electronic supplementary material

Below is the link to the electronic supplementary material.

Supplementary Material 1

## Data Availability

Qualified researchers may request access to patient level data and related study documents including the clinical study report, study protocol with any amendments, blank case report form, statistical analysis plan and dataset specifications. Patient level data will be anonymised, and study documents will be redacted to protect the privacy of our trial participants. Further details on Sanofi’s data sharing criteria, eligible studies, and process for requesting access can be found at: https://www.vivli.org/.
