# Supplementary material for: Efficacy and safety of isatuximab monotherapy to treat relapsed or refractory multiple myeloma: a pooled analysis of clinical trials
Source: Ann Hematol. 2025 Apr 21;104(4):2337–50. doi: 10.1007/s00277-025-06343-9 (PMC12053118; doi:10.1007/s00277-025-06343-9)
Supplement: Supplementary file 1 — Supplementary Material 1 [file 277_2025_6343_MOESM1_ESM.docx]

**Efficacy and safety of isatuximab monotherapy to treat relapsed or refractory multiple myeloma: a pooled analysis of clinical trials**

**Authors:** Meletios Dimopoulos^1, 2^, Kazutaka Sunami^3^, Xavier Leleu^4^, Ravi Vij^5^, Cristina Gasparetto^6^, Kenshi Suzuki^7^, Sandrine Macé^8^, Keisuke Tada^9^, Mutsumi Hirakawa^10^, Shinsuke Iida^11^

**Affiliations:** ^1^Department of Clinical Therapeutics, School of Medicine, National and Kapodistrian University of Athens, Athens, Greece; ^2^Department of Medicine, Korea University, Seoul, South Korea; ^3^Department of Hematology, NHO Okayama Medical Center, Okayama, Japan; ^4^CIC 1082, U1313, CHU, University, Poitiers, France; ^5^Division of Medical Oncology, Washington University in St. Louis, St. Louis, MO, USA; ^6^Hematologic Malignancies and Cellular Therapy, Duke University Medical Center, Durham, NC, USA; ^7^Myeloma/Amyloidosis Center, Japanese Red Cross Medical Center, Tokyo, Japan; ^8^Research and Development, Sanofi, Paris, France; ^9^Research and Development, Sanofi K.K., Tokyo, Japan; ^10^Oncology Medical, Sanofi K.K., Tokyo, Japan; ^11^Department of Hematology and Oncology, Nagoya City University Institute of Medical and Pharmaceutical Sciences, Nagoya, Japan.

**Corresponding author:** Shinsuke Iida, Department of Hematology and Oncology, Nagoya City University Institute of Medical and Pharmaceutical Sciences, Kawasaki 1, Mizuno-cho, Mizuno-ku, Nagoya City, Aichi 467-8601, Japan. Tel: +81-52-853-8738; Fax: +81-52-853-8740; Email: iida@med.nagoya-cu.ac.jp

# Supplementary Information

## Supplementary Table S1

Baseline characteristics of responders (n=44) and non-responders (n=123) to isatuximab monotherapy.

|  | **Responders (n=44)** | **Non-responders (n=123)** |
| --- | --- | --- |
| Age group, n (%) |  |  |
| ≥65–74 years | 13 (29.5) | 53 (43.1) |
| ≥75 years | 15 (34.1) | 20 (16.3) |
| ECOG PS, n (%) |  |  |
| 0 | 22 (50.0) | 51 (41.5) |
| 1 | 19 (43.2) | 60 (48.8) |
| 2 | 3 (6.8) | 12 (9.8) |
| ISS stage, n (%) |  |  |
| I | 19 (43.2) | 34 (27.6) |
| II | 17 (38.6) | 38 (30.9) |
| III | 8 (18.2) | 51 (41.5) |
| Bone marrow plasma cells, n (%) |  |  |
| 0–5% | 3 (6.8) | 18 (14.6) |
| ≥5–20% | 14 (32.8) | 29 (23.6) |
| ≥20–50% | 15 (34.1) | 27 (22.0) |
| ≥50% | 6 (13.6) | 30 (24.4) |
| Unknown/missing data | 6 (13.6) | 19 (15.4) |
| Plasmacytoma,^a^ n (%) | 3 (6.8) | 29 (23.6) |
| Cytogenetics by FISH, n (%) |  |  |
| Standard risk | 30 (68.2) | 61 (49.6) |
| High risk | 7 (15.9) | 36 (29.3) |
| Unknown/missing data | 7 (15.9) | 26 (21.1) |
| eGFR,^b^ n (%) |  |  |
| ≥50 mL/min/1.73 m^2^ | 35 (79.5) | 91 (74.0) |
| ≥30 to <50 mL/min/1.73 m^2^ | 7 (15.9) | 25 (20.3) |
| ≥15 to <30 mL/min/1.73 m^2^ | 1 (2.3) | 2 (1.6) |
| <15 mL/min/1.73 m^2^ | 0 | 0 |
| Unknown/missing data | 1 (2.3) | 5 (4.1) |

^a^Evaluated by independent review committee.

^b^Renal function was defined as follows: normal: eGFR ≥90 mL/min/1.73 m^2^; mild impairment: eGFR ≥60–90 mL/min/1.73 m^2^; moderate impairment: eGFR ≥30–60 mL/min/1.73 m^2^; severe impairment: eGFR ≥15–30 mL/min/1.73 m^2^; end-stage: eGFR <15 mL/min/1.73 m^2^.

ECOG PS, Eastern Cooperative Oncology Group Performance Status; eGFR, estimated glomerular filtration rate; FISH, fluorescence *in situ* hybridization; ISS, International Staging System.

## Supplementary Table S2

Prognostic factors and hazard ratios according to multivariate analysis of progression-free survival.

|  | **n** | **Events, n (%)** | **Median PFS (95% CI)** | **HR (95% CI)** | **P-value** |
| --- | --- | --- | --- | --- | --- |
| Age |  | | | | |
| <65 years | 66 | 40 (60.6) | 5.6 (3.88, 8.48) | Reference |  |
| 65–74 years | 66 | 46 (69.7) | 3.7 (2.37, 5.55) | 0.721 (0.403, 1.289) | 0.2695 |
| ≥75 years | 35 | 21 (60.0) | 10.2 (4.67, 16.59) | 0.298 (0.143, 0.622) | 0.0013 |
| ISS stage at study entry |  | | | | |
| I | 53 | 32 (60.4) | 8.5 (5.62, 12.55) | 0.351 (0.191, 0.644) | 0.0007 |
| II | 55 | 34 (61.8) | 7.1 (5.55, 10.32) | 0.464 (0.265, 0.811) | 0.0071 |
| III | 59 | 41 (69.5) | 2.4 (1.91, 3.38) | Reference |  |
| Cytogenetics by FISH |  | | | | |
| Unknown/missing | 33 | 21 (63.6) | 4.9 (3.55, 8.48) | 0.447 (0.219, 0.913) | 0.0272 |
| Standard risk | 91 | 55 (60.4) | 7.1 (3.94, 8.64) | 0.390 (0.227, 0.670) | 0.0006 |
| High risk | 43 | 31 (72.1) | 3.0 (2.14, 5.55) | Reference |  |
| Plasmacytoma by IRC |  | | | | |
| Yes | 32 | 26 (81.3) | 2.9 (1.87, 3.75) | Reference |  |
| No | 135 | 81 (60.0) | 7.1 (4.70, 8.51) | 0.289 (0.160, 0.520) | <0.0001 |
| Baseline eGFR |  | | | | |
| <60 mL/min/1.73 m^2^ | 55 | 38 (69.1) | 3.4 (2.07, 4.67) | Reference |  |
| ≥60 mL/min/1.73 m^2^ | 106 | 64 (60.4) | 7.4 (5.55, 8.64) | 0.589 (0.334, 1.037) | 0.0666 |

CI, confidence interval; CP, clinical progression; eGFR, estimated glomerular filtration rate; FISH, fluorescence *in situ* hybridization; IRC, independent review committee; ISS, International Staging System; HR, hazard ratio; PFS, progression-free survival.

## Supplementary Table S3

Prognostic factors and hazard ratios according to multivariate analysis of overall survival.

|  | **n** | **Events, n (%)** | **Median OS (95% CI)** | **HR (95% CI)** | **P-value** |
| --- | --- | --- | --- | --- | --- |
| Age |  | | | | |
| <65 years | 66 | 30 (45.5) | 18.9 (12.06, NR) | Reference |  |
| 65–74 years | 66 | 26 (39.4) | 23.1 (13.60, NR) | 0.417 (0.209, 0.831) | 0.0129 |
| ≥75 years | 35 | 15 (42.9) | 23.9 (14.72, 30.75) | 0.355 (0.161, 0.783) | 0.0103 |
| Ethnicity | | | | | |
| Japanese | 33 | 12 (36.4) | 30.8 (23.85, NR) | 0.550 (0.260, 1.167) | 0.1195 |
| Non-Japanese | 134 | 59 (44.0) | 18.9 (13.60, 23.06) | Reference |  |
| ECOG PS | | | | | |
| 0 or 1 | 152 | 60 (39.5) | 23.1 (18.92, NR) | 0.306 (0.148, 0.634) | 0.0014 |
| 2 | 15 | 11 (73.3) | 10.5 (4.53, 13.27) | Reference |  |
| ISS stage at study entry | | | | | |
| I | 53 | 12 (22.6) | NR (23.85, NR) | 0.215 (0.098, 0.474) | 0.0001 |
| II | 55 | 21 (38.2) | 23.1 (16.49, NR) | 0.324 (0.171, 0.613) | 0.0005 |
| III | 59 | 38 (64.4) | 10.5 (6.74, 16.56) | Reference |  |
| Baseline eGFR | | | | | |
| <60 mL/min/1.73 m^2^ | 55 | 29 (52.7) | 16.5 (12.16, 30.75) | Reference |  |
| ≥60 mL/min/1.73 m^2^ | 106 | 38 (35.8) | 23.9 (18.92, NR) | 0.501 (0.266, 0.943) | 0.0322 |

CI, confidence interval; CP, clinical progression; ECOG PS, Eastern Cooperative Oncology Group Performance Status; eGFR, estimated glomerular filtration rate; FISH, fluorescence *in situ* hybridization; IRC, independent review committee; ISS, International Staging System; NR, not reached; HR, hazard ratio; OS, overall survival.

## Supplementary Table S4

Response to isatuximab monotherapy in participants with baseline estimated glomerular filtration rate <40 or ≥40 mL/min/1.73 m^2^.

|  | eGFR <40 mL/min/1.73 m^2^ (n=9) | eGFR ≥40 mL/min/1.73 m^2^ (n=152) |
| --- | --- | --- |
| Best response, n (%) |  |  |
| CR | 1 (11.1) | 2 (1.3) |
| VGPR | 0 | 15 (9.9) |
| PR | 1 (11.1) | 24 (15.8) |
| ORR, n (%) | 2 (22.2) | 41 (27.0) |
| VGPR or better, n (%) | 2 (22.2) | 17 (11.2) |
| Median PFS (95% CI), months | 2.1 (1.9, NR) | 5.6 (4.6, 7.7) |
| Median OS (95% CI), months | 13.9 (4.1, NR) | 23.1 (16.6, NR) |

CI, confidence interval; CR, complete response; eGFR, estimated glomerular filtration rate; NR, not reached; ORR, overall response rate; OS, overall survival; PFS, progression-free survival; PR, partial response; VGPR, very good partial response.

## Supplementary Table S5

Renal response during isatuximab monotherapy in the efficacy analysis population (n=167).

|  | **All (N=167)** |
| --- | --- |
| Baseline eGFR <50 mL/min/1.73 m^2^ | n=35 |
| CR renal,^a^ n (%) | 9 (25.7) |
| Baseline eGFR ≥15–30 mL/min/1.73 m^2^ | n=3 |
| MR renal,^b^ n (%) | 3 (100.0) |

^a^eGFR <50 mL/min/1.73 m^2^ at baseline and ≥1 assessment ≥60 mL/min/1.73 m^2^ during treatment.

^b^eGFR ≥15–30 mL/min/1.73 m^2^ at baseline and ≥1 assessment ≥30–60 mL/min/1.73 m^2^ during treatment.

CR, complete response; eGFR, estimated glomerular filtration rate; MR, minimal response; PR, partial response.

## Supplementary Fig S1

Diagram of the safety and efficacy analysis population sets included in this pooled analysis. Source studies (TED 10893, TED 14905, TED 14154 and TED 14906) [1-5].


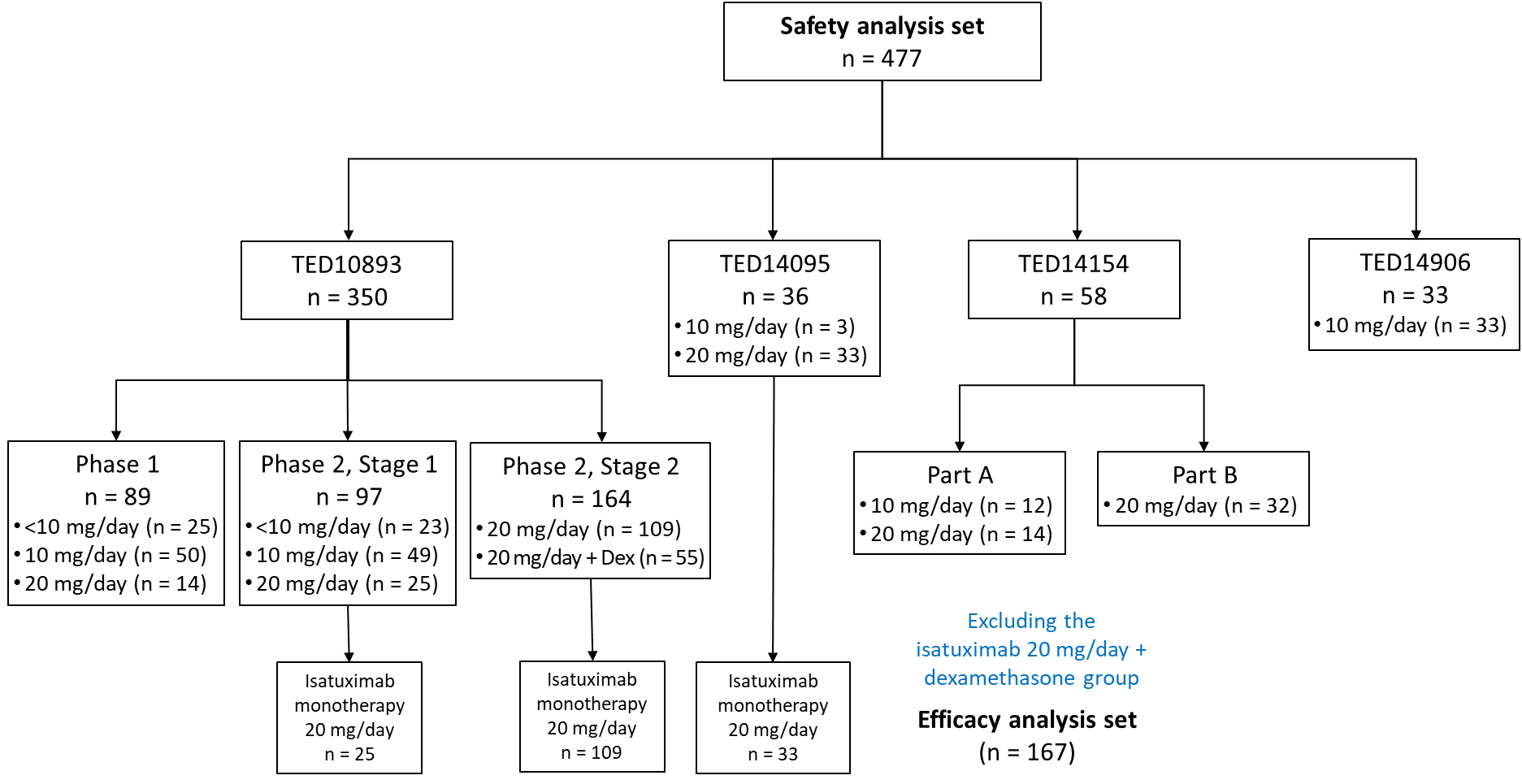


## Supplementary Fig S2

Kaplan-Meier plot for progression-free survival by clinical progression at baseline in the efficacy population (n=167). PFS by CP status was based on three of the IMWG uniform response criteria for clinical relapse, whereby patients meeting any one of the following IMWG criteria during screening were classified as having CP: soft tissue plasmacytoma or occurrence of bone lesions; hypercalcemia (>11.5 mg/dL; >2.875 mM/L); haemoglobin (Hb) <10 g/dL [6]. Patients not meeting any of these criteria were classified as having non-CP.


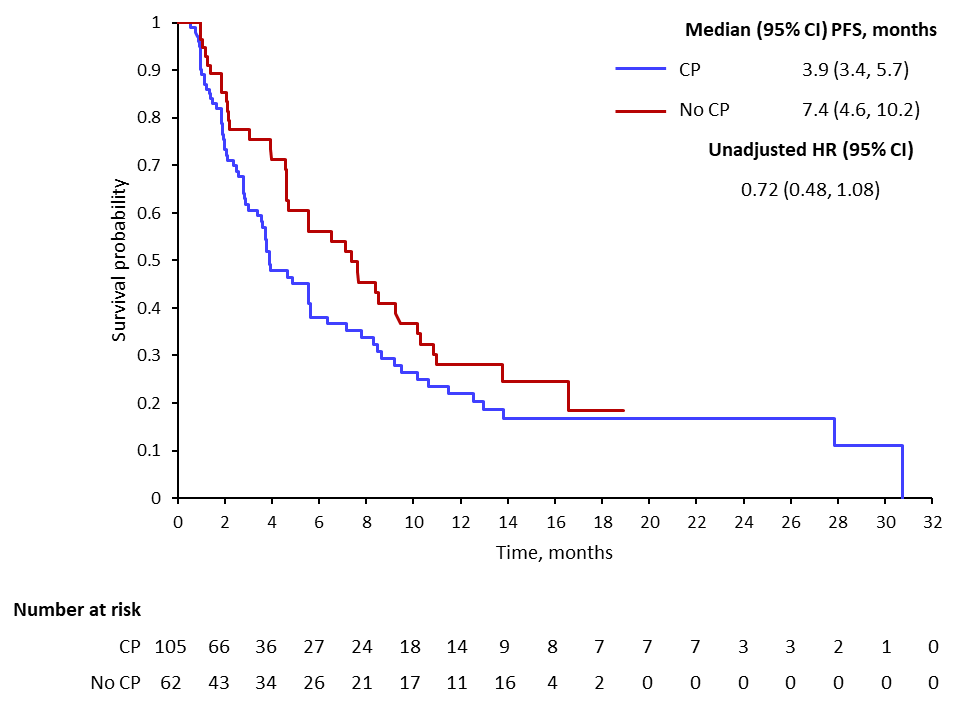


CI, confidence interval; CP, clinical progression; HR, hazard ratio; NR, not reached; PFS, progression-free survival.

## Supplementary Fig S3

Kaplan-Meier plot for overall survival by clinical progression at baseline in the efficacy population (n=167). OS by CP status was based on three of the IMWG uniform response criteria for clinical relapse, whereby patients meeting any one of the following IMWG criteria during screening were classified as having CP: soft tissue plasmacytoma or occurrence of bone lesions; hypercalcemia (>11.5 mg/dL; >2.875 mM/L); haemoglobin (Hb) <10 g/dL [6]. Patients not meeting any of these criteria were classified as having non-CP.


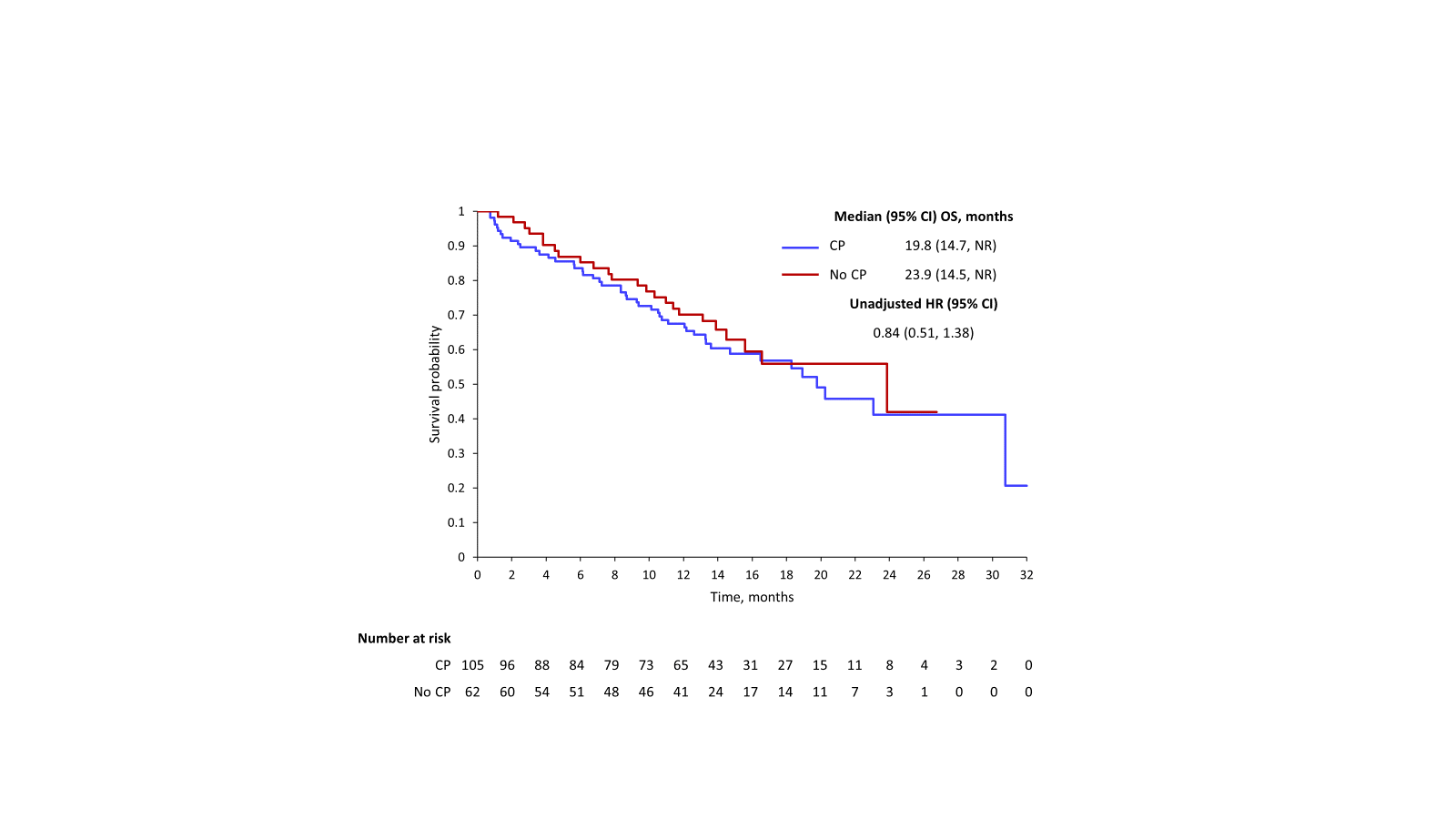


CI, confidence interval; CP, clinical progression; HR, hazard ratio; NR, not reached; OS, overall survival.

**References**

1. Dimopoulos M, Bringhen S, Anttila P, Capra M, Cavo M, Cole C, Gasparetto C, Hungria V, Jenner M, Vorobyev V, Ruiz EY, Yin JY, Saleem R, Hellet M, Macé S, Paiva B, Vij R (2021) Isatuximab as monotherapy and combined with dexamethasone in patients with relapsed/refractory multiple myeloma. Blood 137:1154-1165. doi:10.1182/blood.2020008209

2. Lesokhin A, LeBlanc R, Dimopoulos MA, Capra M, Carlo-Stella C, Karlin L, Castilloux JF, Forsberg P, Parmar G, Tosikyan A, Pour L, Ribrag V, Ribolla R, Abdallah AO, Le Roux N, Dong L, van de Velde H, Mayrargue L, Lepine L, Mace S, Moreau P (2023) Isatuximab in combination with cemiplimab in patients with relapsed/refractory multiple myeloma: A phase 1/2 study. Cancer Med 12:10254-10266. doi:10.1002/cam4.5753

3. Mikhael J, Belhadj-Merzoug K, Hulin C, Vincent L, Moreau P, Gasparetto C, Pour L, Spicka I, Vij R, Zonder J, Atanackovic D, Gabrail N, Martin TG, Perrot A, Bensfia S, Weng Q, Brillac C, Semiond D, Macé S, Corzo KP, Leleu X (2021) A phase 2 study of isatuximab monotherapy in patients with multiple myeloma who are refractory to daratumumab. Blood Cancer J 11:89. doi:10.1038/s41408-021-00478-4

4. Mikhael J, Richter J, Vij R, Cole C, Zonder J, Kaufman JL, Bensinger W, Dimopoulos M, Lendvai N, Hari P, Ocio EM, Gasparetto C, Kumar S, Oprea C, Chiron M, Brillac C, Charpentier E, San-Miguel J, Martin T (2020) A dose-finding Phase 2 study of single agent isatuximab (anti-CD38 mAb) in relapsed/refractory multiple myeloma. Leukemia 34:3298-3309. doi:10.1038/s41375-020-0857-2

5. Sunami K, Suzuki K, Ri M, Matsumoto M, Shimazaki C, Asaoku H, Shibayama H, Ishizawa K, Takamatsu H, Ikeda T, Maruyama D, Kaneko H, Uchiyama M, Kiguchi T, Iyama S, Murakami H, Takahashi K, Tada K, Macé S, Guillemin-Paveau H, Iida S (2020) Isatuximab monotherapy in relapsed/refractory multiple myeloma: a Japanese, multicenter, phase 1/2, safety and efficacy study. Cancer Sci 111:4526-4539. doi:10.1111/cas.14657

6. Rajkumar SV, Harousseau JL, Durie B, Anderson KC, Dimopoulos M, Kyle R, Blade J, Richardson P, Orlowski R, Siegel D, Jagannath S, Facon T, Avet-Loiseau H, Lonial S, Palumbo A, Zonder J, Ludwig H, Vesole D, Sezer O, Munshi NC, San Miguel J, International Myeloma Workshop Consensus P (2011) Consensus recommendations for the uniform reporting of clinical trials: report of the International Myeloma Workshop Consensus Panel 1. Blood 117:4691-4695. doi:10.1182/blood-2010-10-299487
